# Parental Education, Household Income, and Cortical Surface Area among 9–10 Years Old Children: Minorities’ Diminished Returns

**DOI:** 10.3390/brainsci10120956

**Published:** 2020-12-09

**Authors:** Shervin Assari

**Affiliations:** 1 Department of Urban Public Health, Charles R Drew University of Medicine and Science, Los Angeles, CA 92697, USA; assari@umich.edu; 2 Department of Family Medicine, Charles R Drew University of Medicine and Science, Los Angeles, CA 92697, USA

**Keywords:** cortex, children, MRI, brain development, socioeconomic factors, population groups

## Abstract

*Introduction:* Although the effects of parental education and household income on children’s brain development are well established, less is known about possible variation in these effects across diverse racial and ethnic groups. According to the Minorities’ Diminished Returns (MDRs) phenomenon, due to structural racism, social stratification, and residential segregation, parental educational attainment and household income show weaker effects for non-White than White children. *Purpose:* Built on the MDRs framework and conceptualizing race as a social rather than a biological factor, this study explored racial and ethnic variation in the magnitude of the effects of parental education and household income on children’s whole-brain cortical surface area. *Methods:* For this cross-sectional study, we used baseline socioeconomic and structural magnetic resonance imaging (sMRI) data of the Adolescent Brain Cognitive Development (ABCD) study. Our analytical sample was 10,262 American children between ages 9 and 10. The independent variables were parental education and household income. The primary outcome was the children’s whole-brain cortical surface area. Age, sex, and family marital status were covariates. Race and ethnicity were the moderators. We used mixed-effects regression models for data analysis as participants were nested within families and study sites. *Results:* High parental education and household income were associated with larger children’s whole-brain cortical surface area. The effects of high parental education and high household income on children’s whole-brain cortical surface area were modified by race. Compared to White children, Black children showed a diminished return of high parental education on the whole-brain cortical surface area when compared to White children. Asian American children showed weaker effects of household income on the whole-brain cortical surface area when compared to White children. We could not find differential associations between parental education and household income with the whole-brain cortical surface area, when compared to White children, for non-Hispanic and Hispanic children. *Conclusions:* The effects of parental educational attainment and household income on children’s whole-brain cortical surface area are weaker in non-White than White families. Although parental education and income contribute to children’s brain development, these effects are unequal across racial groups.

## 1. Introduction

The brain’s cortical surface area is under the influence of a wide range of factors such as age, sex, socioeconomic status (SES), race, and ethnicity [1,2]. Of all brain regions, the cerebral cortex shows the most evolutionary variation from other mammals as it has evolved most recently [3]. Encompassing 19% of brain neurons [3], the cerebral cortex is last to mature [3,4,5]. The cerebral cortex includes sensory and motor areas. Although the first receives and processes the sensory inputs from all organs, the latter is mainly responsible for controlling voluntary movement [6]. The cerebral cortex has a role in executive function, decision-making, emotion regulation, thought processes, and cognitive function [7]. The cortical surface area has implications for cognitive performance, cognitive reserve, and intelligence [2,8,9,10,11,12,13].

Altered structure and function of the cerebral cortex is linked to a wide range of neurodevelopmental disorders [14,15,16]. Altered cerebral cortex (e.g., surface area, thickness, and volume) is a common finding in neuromuscular diseases [17], depression [18,19], autism spectrum disorder (ASD) [20], obsessive-compulsive disorder (OCD) [21,22], Attention-Deficit/Hyperactivity Disorder (ADHD) [23], and schizophrenia [24].

Alteration of cortical surface area and thickness have both distinct [25,26,27] and overlapping mechanisms [28]. Although they both result in changes in cortical mass (volume), they vastly differ in their underlying biological processes [28]. The altered cortical surface area commonly reflects altered folding and gyrification as a function of the modified progenitor cells’ modified division in the periventricular area during embryogenesis [29,30]. Changes in cortical thickness, however, reflects differences in dendritic arborization and pruning [31]. Regardless of its cause, the whole-brain cortical surface area is a marker of children’s psychopathology and brain development [7].

Research has shown that the development of cerebral cortex is under influence of exposure to stress, disadvantage, drugs, and toxins, which all depend on SES [32,33,34,35]. SES can be regarded as a proxy of exposure to a wide range of environmental factors that influence cortical brain development [32,33,34,35]. These SES effects are beyond the genetic influences on brain development and are mainly environmental, thus preventable. Noble, Sowell, and colleagues documented the positive impact of household income and parental education on brain morphometry [36]. In 1099 typically developing individuals who were 3–20 years old, income was associated with brain morphometry, independent of genetic ancestry. The effect of income on brain cortex surface area, however, was more pronounced for children from lower-income families [36].

Opposite to Noble, Sowell, and colleagues who documented a stronger effect of household income on the brain structure of economically disadvantaged children [36], our previous work has consistently shown opposite patterns. In a recent study using Adolescent Brain Cognitive Development (ABCD) data, the SES effect on amygdala size was less pronounced in Black (socially disadvantaged) than White (socially privileged) children [37]. This observation is in line with our studies showing weaker effects of parental education and household income on trauma [38], ADHD [39], suicide [40], depression [41], anxiety [42], aggression [43], tobacco use [43,44], impulsivity [45], school bonding [46], school performance [47], and inhibitory control [48] for Black than White children. Similar findings are shown in the ABCD [40,48], Add Health [49], Fragile Families and Child Wellbeing Study (FFCWS) [39,45,46,50,51], Monitoring the Future (MTF) [47], National Survey of American Life (NSAL) [41], Flint Adolescents Study (FAS) [42], Early Childhood Longitudinal (ECL) study [52], and the Family and Community Health Study (FACHS) [53,54], all suggesting that parental education and household income provide diminished protection for Black than White families. These findings, which hold across SES indicators, outcomes, settings, age groups, cohorts, and populations, are called Minorities’ Diminished Returns (MDRs).

The MDRs explained above are due to societal rather than behavioral or biological factors. Due to the labor market discrimination, Black families with highly educated parents earn less income and accumulate less wealth over time than White families with identical education and marital status [55]. Black children with highly educated parents are sent to worse schools [52], live in more dangerous environments [56], and have high-risk peers and relatives [57]. In addition, high SES Black children still experience high levels of chronic stress and trauma [38], which is toxic to brain development [58,59,60,61]. Finally, for Black families, SES increases rather than decreases exposure and vulnerability to discrimination.

Conceptualizing race and ethnicity as social rather than biological constructs, we explored racial variation in the magnitude of the effects of parental education and household income on children’s whole-brain cortical surface area. We expected positive associations between parental education and household income with the cortical surface area because parental education and household income are proxies of rich environments with an abundance of intellectually stimulating inputs, all essential for healthy brain development. High parental education and household income reflect positive parenting, economic stability, and lower exposure to stress [62,63,64]. In line with the MDRs framework, we expect the positive effects of parental education and household income on a child’s brain to be smaller in non-White, particularly Black, than White families. We also expect weaker effects for Hispanic than non-Hispanic children.

## 2. Methods

### 2.1. Design and Settings

This is a secondary analysis of existing data. Data were borrowed from the Adolescent Brain Cognitive Development (ABCD) study [65,66,67,68]. The ABCD is a landmark brain development study in the United States. Although detailed information regarding ABCD study methods, sampling, sample, measures, and imaging techniques are available [65,66,67,68,69,70], we briefly review some key aspects of the study.

### 2.2. Participants and Sampling

Participants of the ABCD study were children who were between ages 9 and 11 years. Children in the ABCD study were recruited from multiple cities across states. Overall, participants were enrolled from 21 sites. The primary source of recruitment for the ABCD sample was U.S. school systems. The sampling protocol of the ABCD study is described in detail here [65]. A total number of 10,262 participants entered this analysis. Our analysis’s eligibility included valid data on race, ethnicity, demographics, parental education, parental marital status, household income, and children’s whole-brain cortical surface area. Participants were included in this analysis regardless of their race, ethnicity, or twin status.

### 2.3. Study Variables

The study variables included parental educational attainment and household income (independent variables), parental marital status, and children’s race and ethnicity (moderators), age, sex, and family structure (confounders), and whole-brain cortical surface area (dependent variable).

*Whole-brain cortical surface area.* The outcome was the children’s whole-brain cortical surface area (mm^2^), measured by structural MRI at rest. Our outcome had a normal distribution. To validate our measure, we correlated our outcome as a predictor of Wisconsin cognitive score and Wisconsin fluid cognitive ability in two multilevel regression that controlled for age, sex, race, ethnicity, parental education, household income, parental marital status, as well as site and families (nested data). Model 1 showed a positive association suggesting that our outcome predicts Wisconsin cognitive ability (b = 2.00 × 10^−5^, S.E. = 0.00, t = 8.37, *p* < 0.001; Appendix A) and fluid intelligence quotient (IQ) (b = 5.00 × 10^−5^, t = 7.32, S.E. = 0.00, *p* < 0.001, Appendix A). Although small, positive correlations between the whole-brain cortical surface area and two separate cognitive functioning measures indicate our outcome’s relevance for higher brain function. As such, we conceptualize the higher whole-brain cortical surface area as a positive outcome. Appendix B shows the name of variables in the Data Exploration and Analysis Portal (DEAP).

#### 2.3.1. Moderators

*Race.* Race was identified by the parents. Race was a categorical variable and coded 1 for Black or African American and 0 for White or Caucasian (reference category).

*Ethnicity.* Ethnicity was a dichotomous variable and coded 1 for Latino and 0 for non-Latino (reference category) families.

#### 2.3.2. Independent Variables

*Parental Educational Attainment.* Participants’ education was an ordinal variable: less than high school (reference category), high school, college, and graduate+ school. 

*Household Income.* Household income education was a 3-level nominal variable: less than 50 thousand dollars (reference category), 50–100 thousand dollars, and more than 100 thousand dollars. 

#### 2.3.3. Confounders

Age, sex, ethnicity, and parental marital status were the confounding variables. Parents reported the child’s age and were calculated as months between the date of birth to the date of the study. Sex of the child was a dichotomous variable that was coded 0 for males and 1 for females. Child ethnicity was measured by the self-identification of the parents. Parental marital status was also a dichotomous variable, self-reported by the parent interviewed, and coded 1 vs. 0 for married and unmarried. 

### 2.4. Data Analysis

We used the DEAP for data analysis, provided by the Data Analysis and Informatics Core of ABCD Data Analysis and Exploration Portal (DEAP), which uses R and provides a user-friendly online platform for multivariable analysis of the ABCD data. The DEAP platform is available at: https://deap.nimhda.org. ABCD data were downloaded from: https://nda.nih.gov/abcd. For our univariate analysis, we reported the mean (standard deviation (S.D.)) and frequency (%) depending on the variable type. We also performed ANOVA and Chi-square to compare study variables between racial groups. R square and *p*-value were reported for each model. For each parameter in the model, unstandardized regression coefficients (b), S.E., and the *p*-value were reported. A *p*-value equal to or less than 0.05 was considered as statistically significant.

Linear regression in DEAP is based on mixed-effect models, given participants are tested to families and families are nested to sites. The primary outcome was the children’s whole-brain cortical surface area. The independent variables were parental education, household income, and race. Age, sex, family marital status, and ethnicity were the covariates. As such, in all our models, we controlled for the effects of families as sites. Our multilevel modeling approach is shown in Appendix C. These models were run in a nested fashion, and small variations distinguish them at each step. *Model 1* tested the additive effects of household income, parental education, and race, with the same covariates, without interaction terms. *Model 2* tested the interaction between household income and race or ethnicity, and *Model 3* tested the interaction between parental education and race or ethnicity. We reran all these models one time with race and one time with ethnicity as the moderator. Race altered the associations of interest, but ethnicity did not show any interaction, and we did not report the results due to ethnicity as the moderator. We checked a wide range of assumptions, including the normal distribution of our outcome, lack of collinearity between predictors as well as the distribution of errors for our model (Appendix D)

### 2.5. Ethical Aspect

Our secondary analysis was found by the Charles R Drew University of Medicine and Science (CDU) Institutional Review Board (IRB) to be exempt from a full IRB review. However, the original ABCD study underwent an Institutional Review Board (IRB) in several institutions, including but not limited to the University of California, San Diego (UCSD). The IRB in multiple institutions approved the study protocol, and all children provided assent and parents signed consent.

## 3. Results

### 3.1. Sample Descriptive Data

Table 1 shows descriptive data, overall. This study included 10,262 children who were either 9 or 10 years old. From this number, 5363 (52.3%) were male and 4899 (47.7%) were female. Overall, 6832 (66.6%) were White, 1482 (14.4%) were Black, 221 (2.2%) were Asian American, and 1727 (16.8%) were other/mixed race. Most participants (*n* = 8321; 81.1%) were non-Latino and 1941 (18.9%) were Latino. The mean whole-brain cortical surface area was 186,587.05 mm^2^ (S.D. = 18,313.07 mm^2^). 

Table 1 also compared study variables by race. Parental education and income were both highest in White and lowest in Black families. Other/mixed-race and Asian American families were between White and Black families.

### 3.2. Main Effects

**Figure 1:**
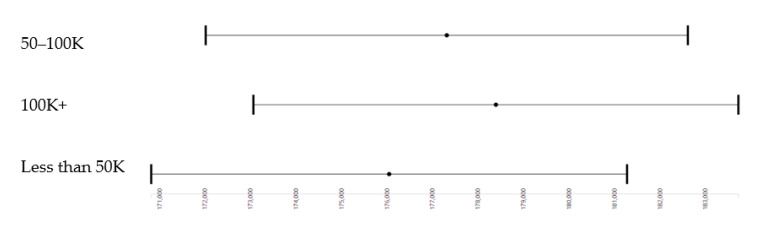
Effects of household income categories on the whole-brain cortical surface area in the whole sample (race, ethnicity, age, sex, parental education, family, and site are controlled).

As shown by Table 2 and Figure 1, income showed a stepwise (dosage-dependent) effect on the whole-brain cortical surface area when all confounders were controlled. These effects were significant for household income of between 50K and 100K (b = 1267.05; *p* = 0.009) as well as income above 100K (b = 2347.38; *p* < 0.001).

**Figure 2:**
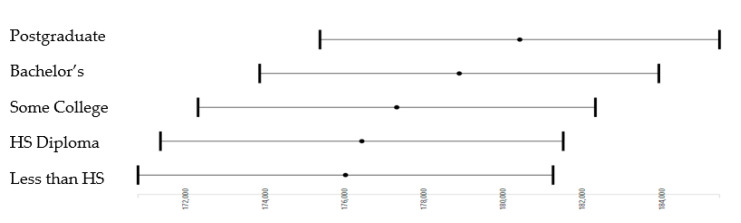
Effects of parental education on the whole-brain cortical surface area in the overall sample. HS: High School.

As shown by Table 3 and Figure 2, parental educational attainment showed a stepwise (dosage-dependent) effect on the whole-brain cortical surface area when all confounders were controlled. These effects were significant for bachelor’s degree (b = 2866.58; *p* = 0.003) or graduate degree (b = 4391.49; *p* < 0.001). There was no advantage in terms of whole-brain cortical surface area when parental education was high school diploma/GED or some college compared to no high school diploma.

### 3.3. Interactive Effects

**Figure 3:**
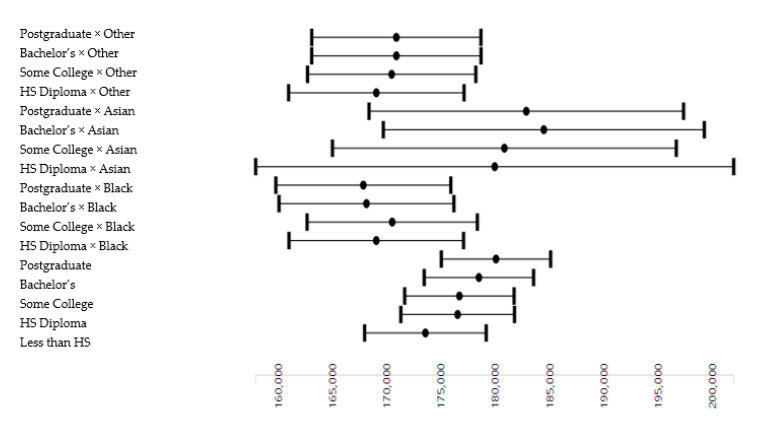
Effects of parental education on the whole-brain cortical surface area by race.

As Table 4 and Figure 3 show, we also found statistical interactions between the effects of parental education categories and race on the whole-brain cortical surface area. These interactions were b = −4525.199, *p* = 0.048 for high school diploma/GED × Black, b = −5429.598, *p* = 0.016 for bachelor’s × Black, and b = −5720.236, *p* = 0.011 for postgraduate × Black. These suggest that the gain in terms of the whole-brain cortical surface area from parental education is diminished for Black than White children. None of the interactions were significant for Asian American or mixed/other race, suggesting that children who were mixed-race or were Asian American similarly gained whole-brain cortical surface area from their parental education compared to White children.

**Figure 4:**
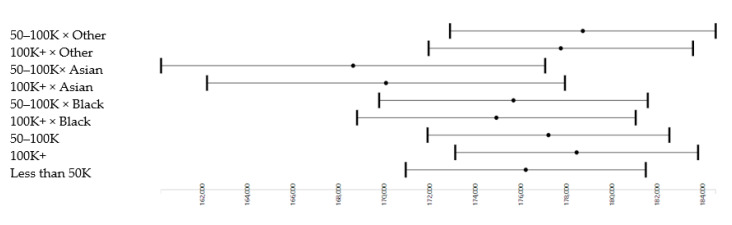
Effects of parental education on the whole-brain cortical surface area in the overall sample.

As shown in Table 5 and Figure 4, we also found interactions between household income and race. Although being Black did not change the effect of household income on the whole-brain cortical surface area, being Asian American interacted with household income, showing the smaller effect of high income for both of these groups. Compared to Whites, Asian Americans showed significantly weaker effects of income between =50K and 100K (b = −7583.14; *p* = 0.022) and income 100+K (b = −6139.97; *p* = 0.035). There was also an unexpected finding. There was a positive rather than negative interaction between income between 50K and 100K × mixed/other race (b = 2507.10, *p* = 0.020).

### 3.4. MDRs Due to Ethnicity

We ran all these models for ethnicity as the moderator as well. Ethnicity showed a significant interaction with parental education or household income on the whole-brain cortical surface area in the whole sample in none of our models. As such, we did not report the results for ethnic variation in our associations of interest. 

## 4. Discussion

We found boosting effects of parental education and household income on children’s whole-brain cortical surface area. At the same time, these effects were weaker for non-Whites than Whites. Parental education’s effect was weaker for Black than White children, household income showed weaker effects for Asian American than White children. Although not consistent, these findings are suggestive of non-Whites’ disadvantage in terms of SES effects on children’s whole-brain cortical surface area, which is also called MDRs. We, however, did not find any evidence suggesting that ethnicity would also alter the parental education or household income on the whole-brain cortical surface area in the whole sample. In other terms, although we observed MDRs based on race, we did not find MDRs due to ethnicity.

The result that high parental education and household income are associated with larger whole-brain cortical surface area is in line with the results of previous research on the salience of family SES as a major social determinant of children’s brain development [62]. Many investigators such as Farah [62], Noble [71], and Lawson [72] have shown an association between SES and various aspects of brain development. For example, Lawson used structural MRI and SES data from a sample of 283 healthy children from the Study of Normal Brain Development and established a positive link between family SES and prefrontal cortical thickness in children. After confounders were controlled and multiple comparisons were adjusted in their study, parental education significantly predicted cortical thickness in the left superior frontal gyrus and right anterior cingulate gyrus. They argued that some cortical changes may mediate (explain) the SES effects on cognitive function of healthy, typically developing children [72]. Sowell has also conducted multiple studies on the developmental change of the cortex [73,74]. She has also studied cortical correlation with demographic factors [75], psychopathologies [76], and environmental factors [77]. Most recently, Sowell used data of 9712 9- and 10-year-old children and showed a stronger negative association of living in high-lead-risk census tracts with brain function and structure in lower SES families compared to higher SES families. Increased lead exposure was associated with smaller cortical surface area, smaller cortical volume, and lower cognitive test scores, but these effects were all more pronounced for low SES families. Based on her findings, a reduction in neighborhood lead exposures may generate a larger gain in terms of boost in brain development for low SES than high SES children [78].

However, our study provided more nuanced data on parental education’s threshold effects and household income on 9–10 years old children’s whole-brain cortical surface area. As Figure 1 shows, we found that although there was a stepwise increase in the whole-brain cortical surface area as parental education increased. A significant difference only exists between the lowest parental education (reference category), which refers to children with parents without a high school diploma and the two highest education categories, meaning that to make a significant difference in our outcome, there was a need for bachelor or graduate-level education, which would be associated with a higher whole-brain cortical surface area of the children compared to those without a high school diploma. Children whose parents had a high school diploma did not show a higher whole-brain cortical surface area than those with parents who did not complete high school. However, as Figure 2 shows, for income, compared to less than 50K, 50+K, or 100+K are associated with the whole-brain cortical surface area. Therefore, the threshold effect that could be observed for parental education was absent for income. In other terms, although high parental education may or may not be associated with some gains in the outcome, depending on how much education attainment is, an increase in income is associated with an increase in whole-brain cortical surface area. This is another example that income may be a more efficient social determinant of health (SDoH) for improving populations’ brain structure, at least in cortical surface areas. The reason income may be more effective is because fewer barriers interfere with the health effects of income.

On the other hand, however, the labor market or high unemployment rate may reduce education’s health effects in some situations [79,80]. This finding is important because it suggests income redistribution by the income tax credit and providing the population with access to cash when they are in economic hardship may be more effective policies than enhancing the proportion of the population that completes high school but never completes college [81]. However, we should acknowledge that income redistribution policies such as higher minimum wage have high proponents in the U.S. as they are found un-American and interference of the government with the free-market [81,82].

Our second finding that education may be associated with a smaller increase in Black’s whole-brain cortical surface area than White children is an extension of the MDRs literature. Our past research shows the same results for aggression [43], tobacco use [44], school attachment [46], school performance [47], ADHD [39], impulsivity [45], stress [38,50], obesity [51], physical health [43], depression [41], and anxiety [42]. For all these outcomes, SES effects are weaker for Black than White children.

These patterns are attributed to a wide range of societal and structural factors. In other words, the MDRs framework argues that structural racism and social stratification are the main reasons family-level SES shows diminished returns for Black than White families [83]. As long as higher-level barriers hinder Black families and as long as discrimination is high for all Black families [84,85,86], and as long as Black families pay extra cost for their upward social mobility [87], high SES will continue to show smaller protective effects for Black than White children.

Other example mechanisms for MDRs also exist. The first mechanism is that high SES Black parents report high stress levels at their occupations [88]. Multiple studies have shown that high SES Black parents earn less income and generate less wealth than their White counterparts with the same education [89]. This is partly because educational and personal decisions have weaker economic consequences in Black than White families [90]. As a result of labor market discrimination [91], high educated Black families experience financial hardship and remain at risk of poverty [55].

Black parents with higher education report higher exposure to second-hand tobacco at work [92]. Spanking [50] and trauma [38] remain high in high SES Black families. At the same time, high SES Black children remain at risk for discrimination [53,54,93]. In fact, high SES children seem to be very vulnerable to discrimination [93]. Simultaneously, highly educated Black families remain in poor neighborhoods with gang problems and exposure to tobacco advertisements [94]. High SES Black families also have high levels of exposure to toxins at home [95]. As a result of the nonequivalence of SES for Black and White families [96], Black children show undesired outcomes even when their parents have high SES [43].

Many SES indicators show weaker effects in changing the living conditions of Black than White families. In a study, Black children from highly SES families were sent to worse schools than Whites with high SES [52]. As a result, high SES Black children still have high-risk peers, which is different from high SES White children [52]. Similarly, Blacks from high SES families reported more substance user family members than high SES White children [57]. Another observation was that high SES Black children have lower literacy about the risk associated with tobacco use risks. However, White children from high SES families have the highest knowledge about tobacco risk [97]. All these reduce the benefit of SES for Black than White families [39,45,46,50,51]. As a result, high SES Black children remain at risk of mental health problems, and high SES White children do not show such patterns [43].

Interestingly enough, MDRs were not specific to Blacks as we found MDRs of household income for Asian American and mixed/other race children. However, we did not find any MDRs due to ethnicity, as Hispanic and non-Hispanic children showed a similar association between parental education or household income on the whole-brain cortical surface area. The literature also shows that MDRs are more prominent for Black [83] than any other racial and ethnic groups, and only a few studies have ever shown MDRs for Latino [43], Asian American [98], and Native American [99] families. Thus, although not just Blacks but all minority groups may face diminishing returns of their SDoHs, these patterns are probably more consequential for Blacks than Latinos. Diminishing returns are shown for LGBT [100], immigrants [101], and Whites living in poor neighborhoods [49], suggesting that MDRs may even happen for minorities without a visible (skin color) or an audible (accent) marker but even invisible sources of marginalization [49]. As marginalized Whites have shown the same patterns, and we know that Whites are not commonly discriminated against, these MDRs’ mechanism is believed to be place-based (contextual) rather than interpersonal discrimination [49].

This study is not the only work that documents the worse-than-expected development of high SES children. Although in our study, racism and social stratification are probable causes of such counterintuitive finding, some related pattern is also shown in high White and Asian American children in high-achieving families. An extensive body of work by Luthar and colleagues [102,103,104,105] has shown elevated rates of behavioral problems such as substance use and even affective problems in White adolescents in affluent settings, wealthy suburbs, high-achieving schools, and high SES families. In several original articles and a review article published in *American Psychologist* [103], it was explained that the potential mechanisms by which students in affluent settings, high-income neighborhoods, and high-achieving schools may in fact be at risk. Given the work by Luthar et al., high SES Black children are not the only group that shows worse-than-expected developmental, emotional, and behavioral trajectories [102,103,104,105]. However, the explanation for worse-than-expected outcomes of White and Asian American children is not related to racism but probably expectations due to high demand, expectations, and aspirations [106]. A study by Trim and Chassin showed that among children of alcoholics (COAs), lower neighborhood SES, and among non-COAs, higher neighborhood SES are associated with higher rates of alcohol use and associated problems [107]. 

### 4.1. Limitations

A few limitations should be listed. This study investigated the cortical surface area, not other aspects of the cortex (e.g., thickness or volume). We also investigated the whole-brain cortex without studying the asymmetry between the right and left hemispheres. We also did not study the surface of specific cortical regions. We also did not investigate the functional aspects of the cortex using fMRI. Finally, we did not measure cortical diffusivity using dMRI. All these areas could possibly generate different results compared to what was reported here.

### 4.2. Future Research

This study only described and did not seek the societal or familial causes of MDRs. Thus, there is a need to explore these MDRs across contexts to test if they are robust across all neighborhoods or are more strongly seen in specific social and physical contexts. Residential and school segregation, neighborhood SES and crime, and environmental toxins may explain MDRs of SES for Black families. Future research may also investigate if discrimination and quality of the school and neighborhood risk reduce the benefits of SES for Black children. We also need to replicate these findings across other marginalizing identities, such as sexual and gender identity, immigration, and place-based marginalization. Finally, there is a need to know what public and social policies can minimize the observed MDRs.

There are several important questions to be considered, e.g., what are similarities and differences between the findings on Black youth and other ethnic minority youth? If there are differences, how might they be explained? Why did Asians show diminished returns in relation to family income, but not family education? Why would multiracial kids show patterns opposite to those seen among other minority groups? Additionally, as noted earlier, it is critical to also include Hispanic children in these analyses. 

### 4.3. Implications

The effect of parental educational attainment and household income on children’s whole-brain cortical surface area are weaker in non-White than White children; that is, while parental education and income generate health outcomes. Overall, these effects are unequal across racial groups, and non-Whites tend to be at a relative disadvantage than Whites to gain health from their resources. To enhance brain health equity, the elimination of the SDoH gap may not be enough. Future research should investigate the roles of residential segregation, labor market discrimination, and quality of schooling in explaining why non-White children from highly educated and high-income parents remain at risk of poor brain development. There is also a need to study economic and social policies that can reduce such risk for middle-class non-White families.

## 5. Conclusions

Although parental education is associated with a larger whole-brain cortex area for children, this effect is weaker for Black than White children. We also observed diminished returns of household income for Asian American children. Eliminating health inequalities requires efforts beyond equalizing SES as we need to equalize the return of SES across social groups.

## Figures and Tables

**Table 1:** Descriptive statistics in the pooled sample by race.

Level	All	White	Black	Asian American	Other/Mixed	*p*
	*n* = 10,262	*n* = 6832	*n* = 1482	*n* = 221	*n* = 1727	
	*n* (%)	*n* (%)	*n* (%)	*n* (%)	*n* (%)	
Ethnicity						
Non-Hispanic	8321 (81.1)	5674 (83.1)	1408 (95.0)	201 (91.0)	1038 (60.1)	<0.001 *
Hispanic	1941 (18.9)	1158 (16.9)	74 (5.0)	20 (9.0)	689 (39.9)	
Sex						
Female	4899 (47.7)	3217 (47.1)	733 (49.5)	112 (50.7)	837 (48.5)	0.256 *
Male	5363 (52.3)	3615 (52.9)	749 (50.5)	109 (49.3)	890 (51.5)	
Married Family						
No	3119 (30.4)	1402 (20.5)	1037 (70.0)	33 (14.9)	647 (37.5)	<0.001 *
Yes	7143 (69.6)	5430 (79.5)	445 (30.0)	188 (85.1)	1080 (62.5)	
Household Income						
<50K	2948 (28.7)	1252 (18.3)	977 (65.9)	35 (15.8)	684 (39.6)	<0.001 *
≥50K and <100K	2941 (28.7)	2079 (30.4)	329 (22.2)	53 (24.0)	480 (27.8)	
≥100K	4373 (42.6)	3501 (51.2)	176 (11.9)	133 (60.2)	563 (32.6)	
Parental Education						
<High School Diploma	374 (3.6)	143 (2.1)	116 (7.8)	5 (2.3)	110 (6.4)	<0.001 *
High School Diploma	850 (8.3)	326 (4.8)	334 (22.5)	3 (1.4)	187 (10.8)	
Some College	2637 (25.7)	1445 (21.2)	592 (39.9)	18 (8.1)	582 (33.7)	
Bachelor’s	2712 (26.4)	2030 (29.7)	222 (15.0)	59 (26.7)	401 (23.2)	
Postgraduate	3689 (35.9)	2888 (42.3)	218 (14.7)	136 (61.5)	447 (25.9)	
	**Mean (S.D.)**	**Mean (S.D.)**	**Mean (S.D.)**	**Mean (S.D.)**	**Mean (S.D.)**	
Age (Months)	118.97 (7.47)	119.03 (7.49)	118.96 (7.24)	119.44 (7.84)	118.67 (7.52)	0.235 **
Whole-brain cortical surface area (mm^2^)	186,587.05 (183,13.07)	189,085.27 (178,30.43)	175,762.13 (171,18.70)	187,249.30 (16,520.15)	185,908.60 (17,984.12)	<0.001 **

**Notes:** Source: Adolescent Brain Cognitive Development (ABCD) Study; * Chi-square test; ** Analysis of Variance (ANOVA).

**Table 2:** Effects of household income level on the whole-brain cortical surface area.

Characteristics	b	S.E.	t	*p*
Income				
<50K				
≥50K and <100K	1267.05	483.99	2.62	0.009
≥100K	2347.38	546.54	4.30	<0.001

**Notes:** Source: ABCD Study; Mixed-effects regression model is used; All covariates such as race, ethnicity, age, sex, income, family, and site were controlled.

**Table 3:** The effects of educational attainment on the whole-brain cortex surface area in the pooled sample.

Title	b	S.E.	t	*p*
Parental Education				
<High School Diploma				
High School Diploma/GED	411.18	974.67	0.42	0.673
Some College	1290.43	893.37	1.44	0.149
Bachelor’s	2866.58 **	949.67	3.02	0.003
Postgraduate	4391.49 ***	961.32	4.57	< 0.001

**Notes:** Source: ABCD Study; Mixed-effects regression model is used; All covariates such as race, ethnicity, age, sex, income, family, and site were controlled; Outcome: whole-brain cortex surface area; ** *p* < 0.01, *** *p* < 0.001.

**Table 4:** Interaction between parental education and race on the whole-brain cortical surface area.

Characteristics	b	S.E.	t	*p*
Sex	16,438.91 ***	289.86	56.71	<0.001
Age	3.39	18.68	0.18	0.856
Hispanic	−2375.15 ***	490.20	−4.85	<0.001
Married	595.55	408.13	1.46	0.144
Income				
< 50K				
≥50K and <100K	1321.45 **	486.56	2.72	0.007
≥100K	2337.38 ***	548.10	4.26	<0.001
Education				
Less than High School Diploma	-			
High School Diploma/GED	2974.74 #	1550.21	1.92	0.055
Some College	3135.11 *	1386.49	2.26	0.024
Bachelor’s	4929.80 ***	1409.24	3.50	<0.001
Postgraduate	6504.50 ***	1410.35	4.61	<0.001
Race				
White	-			
Black	−6334.77 **	1977.91	−3.20	0.001
Asian	−14,335.49 *	6453.45	−2.22	0.026
Other/Mixed	511.17	1909.14	0.27	0.789
2 × 2 Interactions				
High School Diploma/GED × Black	−4525.20 *	2285.52	−1.98	0.048
Some College × Black	−3056.93	2098.03	−1.46	0.145
Bachelor’s × Black	−5429.60 *	2254.01	−2.41	0.016
Postgraduate × Black	−5720.24 *	2252.90	−2.54	0.011
High School Diploma/GED × Asian American	6386.63	10,734.14	0.59	0.552
Some College × Asian American	7278.74	7348.76	0.99	0.322
Bachelor’s × Asian American	10,912.54	6750.42	1.62	0.106
Postgraduate × Asian American	9298.08	6589.36	1.41	0.158
High School Diploma/GED × Other/Mixed-Race	−4519.84	2368.99	−1.91	0.056
Some College × Other/Mixed-Race	−3107.06 #	2053.75	−1.51	0.130
Bachelor’s × Other/Mixed-Race	−2674.71	2093.12	−1.28	0.201
Postgraduate × Other/Mixed-Race	−2676.78	2067.34	−1.29	0.195

# *p* < 0.1, * *p* < 0.05, ** *p* < 0.01, *** *p* < 0.001.

**Table 5:** Interaction between race and household income on children’s whole-brain cortical surface area.

Estimate	b	S.E.	t	*p*
Sex	16,434.88 ***	289.76	56.72	<0.001
Age	2.25	18.67	0.12	0.904
Hispanic	−2539.44 ***	488.71	−5.20	<0.001
Married	554.52	407.95	1.36	0.174
Education				
Less than High School Diploma				
High School Diploma/GED	332.76	974.84	0.34	0.733
Some College	1230.71	893.89	1.38	0.169
Bachelor’s	2775.06 **	950.97	2.92	0.003
Postgraduate	4325.67 ***	962.23	4.50	<0.001
Income				
<50K				
≥50K and < 100K	997.13 #	605.33	1.65	0.100
≥100K	2235.76 ***	632.30	3.54	<0.001
Race				
White				
Black	−10,161.87 ***	723.10	−14.05	<0.001
Asian American	465.89	2590.25	0.18	0.857
Other/Mixed	−3598.87 ***	741.82	−4.85	0.000
Income ≥ 50K and < 100K × Black	−540.45	1152.50	−0.47	0.639
Income ≥ 100K × Black	−1291.51	1399.77	−0.92	0.356
Income ≥ 50K and < 100K × Asian American	−7583.14 *	3308.44	−2.29	0.022
Income ≥ 100K × Asian American	−6139.97 *	2905.36	−2.11	0.035
Income ≥ 50K and < 100K × Mixed/Other Race	2507.10 *	1074.79	2.33	0.020
Income ≥ 100K × Mixed/Other Race	1540.00	1023.69	1.50	0.133

# *p* < 0.1, * *p* < 0.05, ** *p* < 0.01, *** *p* < 0.001.

**Figure A1:**
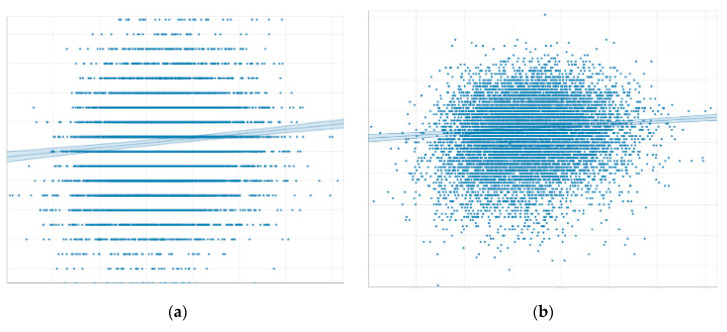
Correlation between whole-brain cortex surface area (our outcome) and our cognitive validation measures. (**a**) Correlation between whole-brain cortex surface area (our outcome) and Wisconsin Cognitive ability (TSS); (**b**) Correlation between whole-brain cortex surface area (our outcome) and National Institutes of Health (NIH) Toolbox Fluid Cognitive ability.

**Figure A2:**
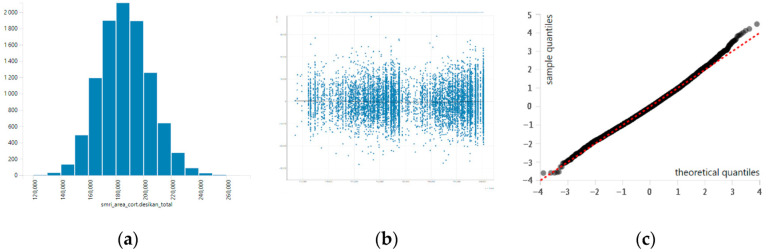
Testing assumptions of our models. (**a**) Histogram showing normal distribution of our outcome, (**b**) residuals of our model, and (**c**) association between observed and theoretical quantiles of our model.

## Appendix B

**Table A1:** Variable in this study.

Construct	Role	Type	Variable Name
Whole-brain cortex surface area	Outcome	Continuous	smri_area_cort.desikan_total
NIH Toolbox Fluid Cognitive ability	Validation of the Outcome	Continuous	nihtbx_fluidcomp_uncorrected
Wisconsin Cognitive ability (TSS)	Validation of the Outcome	Continuous	pea_wiscv_tss
Race	Moderator	Categorical	race.4level
Parental Education	Predictor	Categorical	high.educ.bl
Household Income	Predictor	Categorical	household.income.bl
Age	Covariate	Continuous	age
Sex	Covariate	Categorical	sex
Ethnicity (Hispanic)	Covariate	Categorical	hisp

## Appendix C

**Table A2:** Mixed-effect regression model formulas to test the main effects and interactions between parental education, household income, and race on the whole-brain cortical surface area of American children.

*Model 1*
Whole-brain cortical surface area = Parental Education + Race + Sex + Married Family + Age + Hispanic
*Model 2*
Whole-brain cortical surface area = Household Income + Race + Sex + Married Family + Age + Hispanic
*Model 3*
Whole-brain cortical surface area = Household Income + Parental Education + Race + Sex + Married Family + Age + Hispanic + Parental Education x Race
*Model 4*
Whole-brain cortical surface area = Parental Education + Household Income + Race + Sex + Married Family + Age + Hispanic + Household Income x Race

**All Models:** Random: ~(Site/Family).
